# In Vitro and In Vivo Effects of Docetaxel and Dasatinib in Triple-Negative Breast Cancer: A Research Study

**DOI:** 10.7759/cureus.43534

**Published:** 2023-08-15

**Authors:** Ioannis D Passos, Dimochristos Papadimitriou, Areti Katsouda, Georgios E Papavasileiou, Apostolos Galatas, Panagiotis Tzitzis, Alexandra Mpakosi, Maria Mironidou- Tzouveleki

**Affiliations:** 1 Surgical Department, 219 Mobile Army Surgical Hospital, Didymoteicho, GRC; 2 Laboratory of Clinical Pharmacology, Faculty of Medicine, School of Health Sciences, General Hospital of Thessaloniki "G. Gennimatas" /Aristotle University of Thessaloniki, Thessaloniki, GRC; 3 Faculty of Medicine, School of Health Sciences, Aristotle University of Thessaloniki, Thessaloniki, GRC; 4 1st Department of Obstetrics & Gynaecology, Medical Faculty, Papageorgiou General Hospital/Aristotle University of Thessaloniki, Thessaloniki, GRC; 5 Department of Microbiology, General State Hospital of Nikaia “Saint Panteleimon", Nikaia, GRC; 6 1st Department of Pharmacology, Faculty of Medicine, School of Health Sciences, Aristotle University of Thessaloniki, Thessaloniki, GRC

**Keywords:** triple-negative breast cancer, dasatinib, docetaxel, kinase inhibitors, targeted therapies, chemotherapy

## Abstract

Introduction

Triple-negative breast cancer (TNBC) comprises a heterogeneous group of tumors with a single trait in common: an evident aggressive nature with higher rates of relapse and lower overall survival in the metastatic context when compared to other subtypes of breast cancer. To date, not a single targeted therapy has been approved for the treatment of TNBC, and cytotoxic chemotherapy remains the standard treatment. In the present experimental study, we examine the effects of the chemotherapeutic docetaxel and the bcr/abl kinase inhibitor dasatinib on TNBC cell lines (in vitro) and on TNBC tumor xenograft mouse models (in vivo).

Materials and methods

TNBC cell lines were cultivated and treated with various concentrations of docetaxel and dasatinib (5 nM to 100 nM). Cell death and apoptosis were studied by flow cytometry. TNBC cell lines were then injected in BALB/c athymic nude mice to express the tumor in vivo. Four groups of mice were created (group A: control; group B: DOC; group C: DAS; group D: DOC + DAS) and treated, respectively, with the drugs and their combination. Tumors were obtained, maintained in a 10% formaldehyde solution, embedded in paraffin, and sent for further histological evaluation (hematoxylin-eosin staining and immune-histochemical analysis) to assess the tumor growth inhibition.

Results

The cytotoxic effects of docetaxel seem statistically important, with little effect on apoptosis. The effect of dasatinib in vitro and vivo is statistically important, in terms of apoptosis and tumor reduction, with little adverse effects.

Conclusions

TNBC is a difficult-to-treat oncologic condition, even in an experimental setting. Promising results concerning the addition of targeted therapies (dasatinib) to the conventional cytotoxic ones (docetaxel) have been shown, awaiting further evaluation.

## Introduction

Breast cancer (BC) constitutes a diverse clinical entity in terms of morphology, biological behavior, clinical characteristics, and progression. While BC remains one of the most prevalent neoplasms, its incidence is substantially increasing in the female population, and its mortality has been declining over the last three decades primarily due to i) implementation of screening programs; ii) effective treatment for early-stage BC; and iii) utilization of adjuvant chemotherapy [1]. BC histological classification does not always provide enough information to evaluate the tumor's biological behavior and eventually the therapeutic approach. As a result, a novel and more reliable molecular classification system has been developed to highlight differences between similar carcinomas and assist clinicians in tailoring individualized therapeutic plans and better determining prognosis [1,2]. Subsequently, breast carcinomas are divided into two main categories based on whether they express estrogen receptors (ER+/ER-) or not, while later the overexpression of human epidermal growth factor 2 (HER2) in BC tumors was examined. Most carcinomas expressing both ER and progesterone receptors (PR) present with low malignant potential, whereas tumors that do not express both of these receptors in addition to HER2 are frequently aggressive in nature [1,3-5]

The term triple-negative breast cancer (TNBC) is used to describe BC that lacks the expression of ER, PR, and HER2 [4,5]. TNBC accounts for approximately 15% of all BC and is most common in premenopausal women under the age of 40, women of color, and women of Hispanic and Indian descent [1,5,6]. TNBC is a malignant tumor with a poor prognosis, exhibiting frequent and significant distant metastases, including the spine, liver, lungs, and brain [7]. Furthermore, it is associated with BRCA1 gene mutations in at least 25% of cases [5,8]. TNBC gene expression analysis has revealed six distinct subtypes, including two basic types (BL1 and BL2), one immunomodulatory, one mesenchymal, one mesenchymal stem cell, and one tubular androgen receptor (LAR) [9].

Cytotoxic chemotherapy is currently the mainstay of treatment for TNBC. Taxanes and anthracyclines are initially administered, followed by alkylating agents, antimetabolites, and vinca alkaloids. TNBC is characterized by high-grade chemosensitivity, but also by significant recurrence rates and a poor prognosis [10]. However, the relatively short disease-free interval and the considerable side effects of chemotherapy regimens require the development of new therapeutic medications. New data indicates that prolonging the period of adjuvant chemotherapy in TNBC is associated with very promising outcomes [11]. Potential therapeutic targets for targeted treatment are the surface receptors of cancer cells such as 1) the endothelial receptor (epidermal growth factor receptor, EGFR) or C-KIT); 2) parts of the protein kinase of the MAP kinase signaling pathway (Ras/MAPK) or the B protein kinase (PI3K/Akt); 3) induction of damage to single or double helix DNA damage by specific chemotherapeutic agents; and 4) inhibition of repair of the defective DNA (inhibitors PARP-poly-ADP-ribose polymerase inhibitors) [12]. To date, not a single targeted therapy has been approved for the treatment of TNBC, and cytotoxic chemotherapy remains the gold standard of treatment [13]. The current study will provide insight into the most recent and effective pharmacological interventions for TNBC and will describe and compare the action (and combination) of two specific pharmaceutical agents, the chemotherapeutic agent docetaxel and the tyrosine kinase inhibitor dasatinib, both of which have shown encouraging efficacy in TNBC models in both experimental and early clinical stages. So far, these medications have not been investigated in combination with TNBC.

## Materials and methods

For the completion of the research protocol, as well as the in vitro and in vivo studies, the researchers used TNBC MDA-MB-231 cell lines (ATCC® HTB-26TM, American Type Culture Collection, LGS Standards, Manassas, VA 20108, USA), which derived from a metastatic pleural effusion of TNBC from a 51-year-old woman [14]. Additionally, they used BALB/c laboratory mice (Pasteur Institute, Athens, Greece) [15,16] and the pharmaceutical agents docetaxel (Taxotere®, Sanofi Aventis) and dasatinib (Sprycel®, Bristol Myers Squibb).

The Clinical Pharmacology Laboratory-Department of Medicine/Faculty of Health Sciences of Aristotle University of Thessaloniki Committee for protocol evaluation of laboratory animal studies granted authorization for this experiment under the registration number: EL54-BIOexp-04.

The pharmaceutical agents were kindly provided by the Oncology Department of the “Theageneion” Anticancer Hospital of Thessaloniki, Greece, after the termination of the patients’ treatment plan (change of pharmaceutical agent, end of chemotherapy sessions, excess vials/tablets).

The entire experimental protocol was performed in the Department of Cell Cultures and in the Laboratory of Experimental Surgery of the 1st Department of Pharmacology in the Faculty of Medicine, School of Health Sciences of the Aristotle University of Thessaloniki, while the flow cytometry method was performed with the help of the flow cytometer "Guava® ViaCount easyCyte" (Merck, KGaA, Darmstadt, Germany, EMD Millipore Corporation, Hayward, CA 94545, USA) in the Laboratory of Physiology, in the same faculty.

In vitro study

For the in vitro study, TNBC MDA-MB 231 (American Type Culture Collection, LGS Standards, Manassas, USA) cells were plated in cultures in L-15 Leibowitz medium, with a 10% FBS solution, and plated in six flasks of 75 cm2 [16,17]. Cells were then placed in the incubator at 37°C, in 0.1% CO2, and in serum-free medium for 12-24 hours to allow all cells to be in the same phase of the cell cycle. Every 2-3 days, a passage was performed with fresh medium [18]. The chemotherapeutic drug docetaxel was dissolved in a serum-free medium and at concentrations of 1 nM, 10 nM, 20 nM, 50 nM, and 100 nM. Similarly, the kinase inhibitor dasatinib was dissolved in a serum-free medium and at concentrations of 1 nM, 10 nM, 20 nM, 50 nM, and 100 nM. One flask was used as a control group, and the other five were given docetaxel at the above concentrations. The concentrations were determined by similar corresponding experimental procedures reported in the literature [19]. The cells were then rinsed with PBS to remove dead cells, and the PBS was aspirated with a glass pipette. Cells were lifted from the slides with trypsin, centrifuged, and then measured by flow cytometry at 24 and 48 hours to study cell death and the possible induction of apoptosis [20].

In vivo study

Twenty-four female athymic nude mice (BALB/c, Pasteur Institute, Athens, Greece), aged 6-8 weeks and of 20 g average weight, were divided into four groups of six animals (Group A: Control group; Group B: Docetaxel-DOC administration group; Group C: Dasatinib-DAS administration group; D-Docetaxel group; and Dasatinib-DOC + DAS administration group) [15,18]. The animals were kept in cages with free access to food and water and 12-hour light/dark cycles and handled in accordance with Council Directive 86/609 of the European Communities and Directive 2003/65 of the European Parliament [21,22].

**Figure 1:**
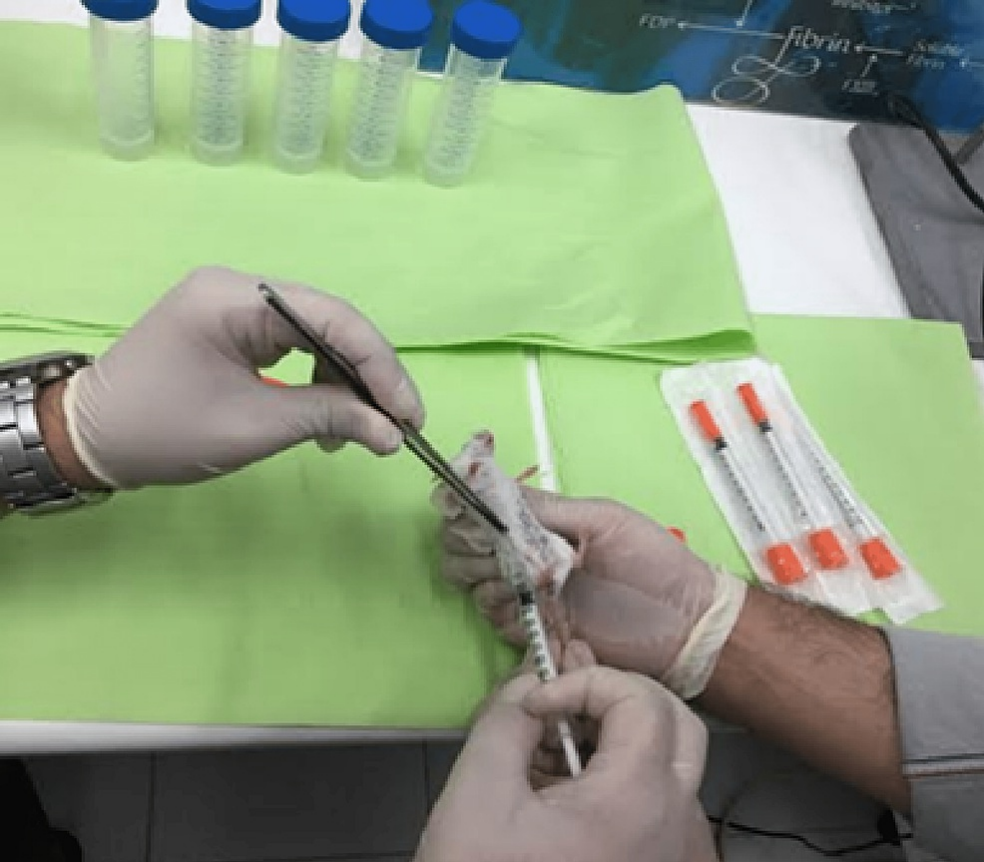
Cell injection. Injection of the cells was performed in the subcutaneous mammary fat of the laboratory animals, and they were observed daily for visible mass development.

Approximately 106 MDA-MB 231 TNBC cells dissolved in 0.2 ml of PBS were administered per female animal using 1 ml and 0.5 ml insulin syringes with a 28G needle diameter. The laboratory animals were temporarily anesthetized after being placed inside a glass cage containing ether, and the cells were injected into their subcutaneous mammary fat (Figure 1) to develop TNBC in vivo.

The animals were observed daily for visible tumor growth and the measurement of the tumor volume developed in their mass gland was calculated with a special caliper in 10 measurements from day one of drug injection [23]. At the same time, their body weight was measured on a special scale on days 1, 3, 6, 9, 11, 13, 16, 19, 22, 25, 28, 31, 33, 36, 40, 47, 50, 53, 56, 59, 62, 65, and 68 for groups A and B. The sacrifice of groups A and B took place on the 69th day. Docetaxel treatment for groups A and B was initiated 33 days after the injection of cancer cells. The body weight of the animals was measured on a special scale on days 1, 2, 5, 8, 11, 14, 17, 20, 23, 26, 29, 32, 35, 39, 42, 48, 51, 54, 57, 60, 63, 66, 69, 72, 75, 78, 81, 84, and 88 for groups C and D, and the volume developed in their mass gland was measured with a special caliper in 10 measurements from day one of the injection of the drug agents [24]. The sacrifice of groups C and D was performed on day 88. Treatment with docetaxel and dasatinib for groups C and D was initiated 42 days after the injection of cancer cells.

**Figure 2:**
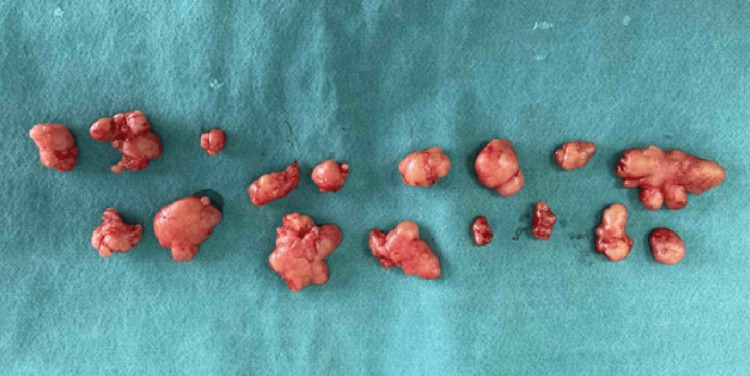
Laboratory animals’ mammary glands. Picture of the laboratory animals’ mammary glands before they were sent for histopathological investigation (magnified picture).

After the laboratory animal sacrifice, the part of their mammary gland corresponding to the injection site of the cancer cells was obtained, placed in a 10% formalin solution, and sent to the Laboratory of Pathological Anatomy of the Medical School of Aristotle University of Thessaloniki for further histopathological investigation (Figure 2).

Statistical analysis

Statistical analysis of the data obtained from both in vitro and in vivo studies was performed using the ToolPak VBA/Statistics program of Microsoft Excel and Statistical Product and Service Solutions (SPSS) software (IBM, Armonk, NY). A t-test for two-sample assuming unequal variances was used. The p-value for statistical significance was defined as 5% (p<0.05).

## Results

In vitro study

Docetaxel at 24 Ηours

**Figure 3:**
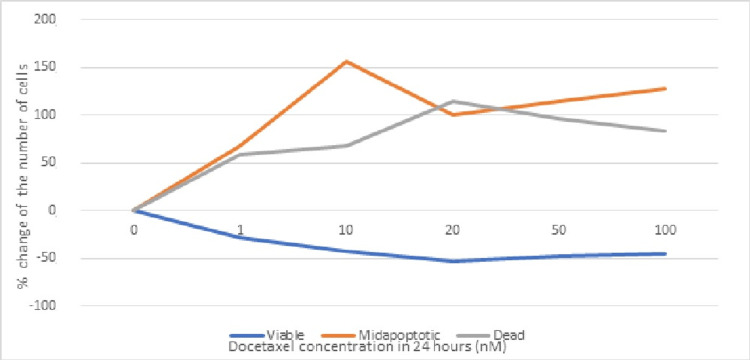
Figure that depicts the change in percentage of the number of cancer cells 24 hours after the administration of docetaxel in various concentrations. nM: nanomoles

The following tables (Tables 1-2) and Figure 3 show the concentrations of the administered docetaxel and the number of viable, apoptotic, and dead cells in percentage (%), 24 hours after administration of different concentrations of docetaxel to TNBC cell lines.

**Table 1:** Docetaxel concentration and its effect on the number of cancer cells (%) in 24 hours. nM: nanomoles, %: percentage

Docetaxel concentration in 24 hours (nM)	Viable cells (%)	Apoptotic cells (%)	Dead cells (%)
C	68	8	24
1	48.5	13.4	38.1
10	39.1	20.5	40.4
20	32.4	16	51.6
50	35.7	17.2	47.1
100	37.8	18.2	44

**Table 2:** Mean value and standard deviation (SD) of percentage of the TNBC cells in various phases (viable/apoptotic/dead cells) 24 hours after the administration of docetaxel in various concentrations. nM: nanomoles, %: percentage

	Viable cells (%)	Apoptotic cells (%)	Dead cells (%)
Mean	43.58	15.55	40.86
SD	13.12	4.38	9.55
2SD	26.24	8.77	19.1

A statistical analysis with the use of a t-test was realized to determine if (whether or not) there is a statistically significant difference in the percentage change of TNBC cells in the "viable" and "dead" phases 24 hours after administration of different concentrations of docetaxel. The test revealed that there is no statistically significant difference in the change of TNBC cell percentage between the "viable" and "dead" phases 24 hours after administration of different concentrations of docetaxel (p=0.69>0.05). Furthermore, statistical analysis to determine if (whether or not) there is a statistically significant difference in the TNBC cell percentage change in the "viable" and "apoptotic" phases 24 hours after administration of different concentrations of docetaxel was realized. The results showed that there is statistical significance between the two groups (p=0.002<0.05).

Based on the above, it appears that the administration of docetaxel to TNBC cell lines induces/activates the process of programmed cell death (apoptosis) 24 hours after administration at a rate that is judged to be statistically significant (p=0.002), at all administered concentrations. Docetaxel also presents high cytotoxicity that is reflected by the increased number of dead cells. Indeed, as the concentrations of administered docetaxel increased, the number of apoptotic and dead cells also increased.

Docetaxel in 48 Hours

**Figure 4:**
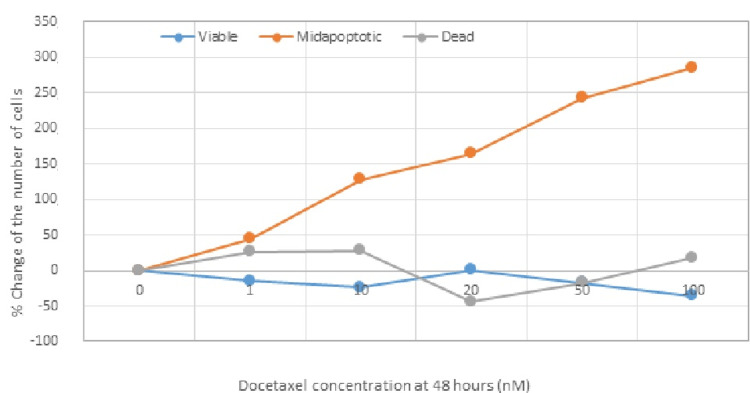
Figure of the percentage (%) of change in the number of cancer cells 48 hours after the administration of docetaxel in different concentrations. nM: nanomoles, %: percentage

The following tables (Tables 3-4) and Figure 4 show the concentrations of the administered docetaxel and the percentage (%) of viable, apoptotic, and dead cells, 48 hours after administration of different concentrations of docetaxel in TNBC cell lines.

**Table 3:** Concentration of administrated docetaxel and the effect on the percentage (%) of TNBC cells at 48 hours. nM: nanomoles, %: percentage

Docetaxel concentration at 48 hours (nM)	Viable cells (%)	Apoptotic cells (%)	Dead cells (%)
C	67	6.8	26.2
1	57.1	9.8	33.1
10	51	15.5	33.5
20	67.3	18	14.7
50	55.1	23.3	21.6
100	43.01	26.2	30.79

**Table 4:** Mean and standard deviation (SD) of the percentage of ΤΝΒC cells at different phases (viable, apoptotic, dead) 48 hours after the administration of different concentrations of docetaxel. %: percentage

	Viable cells (%)	Apoptotic cells (%)	Dead cells (%)
Mean	56.75	16.6	26.64
Standard deviation	9.39	7.51	7.40

Statistical analysis with a t-test estimated the significance of the difference in the percentage of ΤΝΒC cells between the phases “viable” and “dead,” 48 hours after the administration of different concentrations of docetaxel. It reveals a statistically significant difference in change of the percentage of the TNBC cells in phases “viable” and “dead,” 48 hours after the administration of different concentrations of docetaxel (p=0.0001<0.05). Additionally, statistical analysis with a t-test showed that there is a significant difference (p=0.00009<0.05) in the percentage change of TNBC cells in the "viable" and "apoptotic" phases at 48 hours after administration of different concentrations of docetaxel.

Based on the above, it appears that the administration of docetaxel to TNBC cell lines induces/activates the process of programmed cell death (apoptosis) 48 hours after administration at a rate judged to be of high statistical significance (p<0.05), at all administered concentrations. Moreover, the administration of docetaxel at increasing concentrations correspondingly increases the degree of apoptosis at 48 hours, with no significant effect on the number of dead cells. In fact, as the concentrations of docetaxel administered increased, the number of apoptotic cells also increased.

Dasatinib at 24 Hours

**Figure 5:**
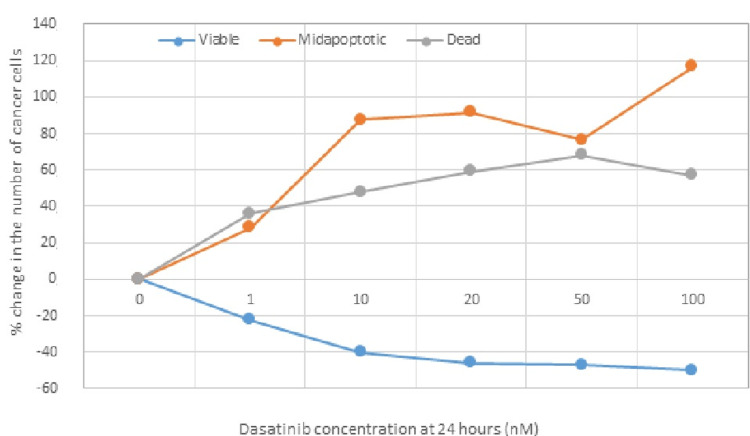
Depiction of the percentage (%) of change in the number of cancer cells 24 hours after the administration of dasatinib in various concentrations.

The following tables (Tables 5-6) and Figure 5 show the concentrations of administered dasatinib and the number of viable, apoptotic, and dead cells in %, 24 hours after administration of different concentrations of dasatinib in TNBC cell lines.

**Table 5:** Dasatinib concentration and the effect on percentage (%) of TNBC cells at 24 hours. nM: nanomoles

Dasatinib concentration at 24 hours (nM)	Viable cells (%)	Apoptotic cells (%)	Dead cells (%)
C	60	12	28
1	46.5	15.4	38.1
10	36.1	22.5	41.4
20	32.4	23	44.6
50	31.7	21.2	47.1
100	30	26	44

**Table 6:** Mean and standard deviation (SD) of the percentage (%) of ΤΝΒC cells in different phases (viable, apoptotic, dead) 24 hours after the administration of different concentrations of dasatinib.

	Viable cells (%)	Apoptotic cells (%)	Dead cells (%)
Mean	39.45	20.01	40.53
Standard deviation (SD)	11.68	5.25	6.86

Statistical analysis of the percentage change of the number of ΤΝΒC cells in phases “viable” and “dead,” 24 hours after the administration of different concentrations of dasatinib with the use of a t-test, resulted in non-significant (p=0.84>0.05) difference of percentage of TNBC cells between the “viable” and “dead” cell categories, 24 hours after the administration of different concentrations of dasatinib. In addition, the statistical analysis of the percentage change of the number of ΤΝΒC cells in phases “viable” and “apoptotic,” 24 hours after the administration of different concentrations of dasatinib, resulted in a highly significant (p=0.007<0.05) difference of percentage of TNBC cells between the “viable” and “apoptotic” cell categories, 24 hours after the administration of different concentrations of dasatinib.

Based on the above, it appears that the administration of dasatinib in TNBC cell lines induces/activates the process of programmed cell death (apoptosis) 24 hours after administration at a rate that is considered statistically significant (p<0.05), at all administered concentrations, while it presents with a moderate degree of cytotoxicity, which is reflected by the increased number of dead cells. Indeed, as the concentrations of administered dasatinib increased, the number of apoptotic and dead cells also increased.

Dasatinib at 48 Hours

**Figure 6:**
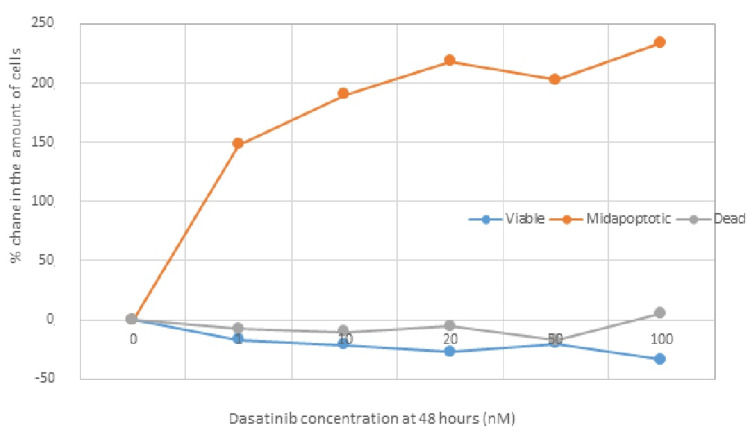
Graphical representation of the percentage change in the amount of cancer cells 48 hours after administration of dasatinib at different concentrations.

The following tables (Tables 7-8) and Figure 6 exhibit the concentrations of administered dasatinib and the number of viable, apoptotic, and dead cells in percentage, 48 hours after administration of different concentrations of dasatinib in TNBC cell lines.

**Table 7:** The concentration of dasatinib at 48 h and its effect on the percentage of cancer cells. nM: nanomoles, %: percentage

Dasatinib concentration at 48 hours (nM)	Viable cells (%)	Apoptotic cells (%)	Dead cells (%)
C	65	8.8	26.2
1	54.1	21.8	34.1
10	51	25.5	23.5
20	47.3	28	24.7
50	51.8	26.6	21.6
100	43.01	29.4	27.59

**Table 8:** The mean and standard deviation (SD) of the percentage of TNBC cells in their different phases (viable, apoptotic, dead) at 48 hours after administration of different concentrations of dasatinib.

	Viable cells (%)	Apoptotic cells (%)	Dead cells (%)
Mean	52.03	23.35	26.28
Standard deviation (SD)	7.44	6.92	3.97

Statistical analysis revealed that there is a statistically significant difference in the percentage (%) change of TNBC cells in the "viable" and "dead" phases 48 hours after administration of different concentrations of dasatinib. The results suggest that there is a highly significant difference (p=0.00008<0.05) between the "viable" and "dead" phases of the cells 48 hours after administration of different concentrations of dasatinib. Additionally, there is a statistically significant difference in the percentage change of TNBC cells in the "viable" and "apoptotic" phases 48 hours after administration of different concentrations of dasatinib (p=0.00005<0.05).

It is obvious that the administration of dasatinib at various (increasing) concentrations induces programmed cell death (apoptosis) 48 hours after administration to a statistically significant degree (p<0.05) with a high degree of cytotoxicity. Indeed, apoptosis increases with increasing concentrations of the administered drug in the cellular tumor lines, with a significant effect on the number of dead/killed cells.

Docetaxel and Dasatinib at 24 Hours

**Figure 7:**
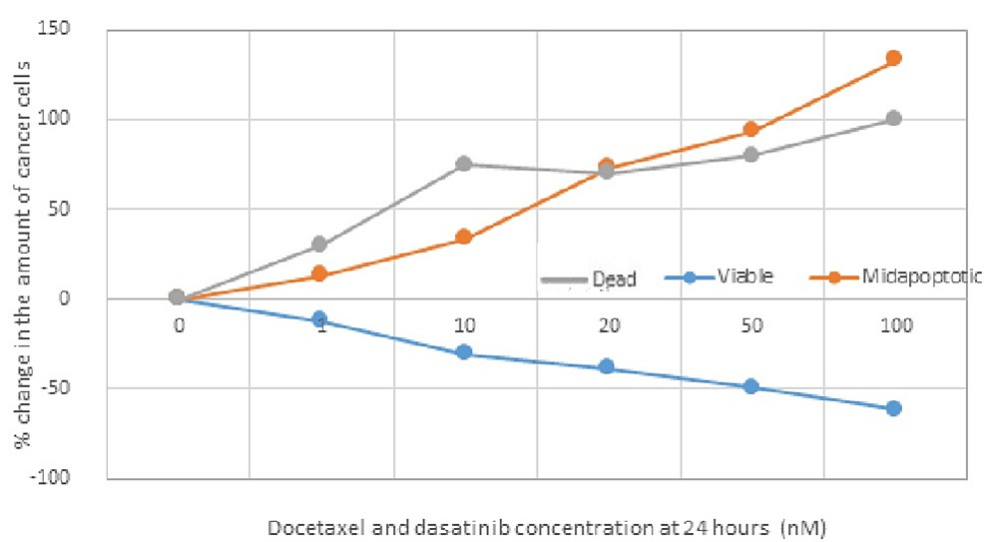
Graphical representation of the percentage (%) change in the amount of cancer cells 24 hours after administration of docetaxel and dasatinib at different concentrations.

The following tables (Tables 9-10) and Figure 7 depict the concentrations of administered docetaxel and dasatinib and the number of viable, apoptotic, and dead cells in percentage (%), 24 hours after administration of different concentrations of these two drug agents in TNBC cell lines.

**Table 9:** The concentrations of docetaxel and dasatinib at 24 hours and their effect on the percentage of the number of cancer cells. nM: nanomoles

Concentration of docetaxel and dasatinib at 24 hours (nM)	Viable cells (%)	Apoptotic cells (%)	Dead cells (%)
C	65	15	20
1	57	17	26
10	45	20	35
20	40	26	34
50	33	29	36
100	25	35	40

**Table 10:** The mean and standard deviation (SD) of the percentage (%) of TNBC cells in their different phases (viable, apoptotic, dead) at 24 hours after co-administration of different concentrations of docetaxel and dasatinib.

	Viable cells (%)	Apoptotic cells (%)	Dead cells (%)
Mean	44.16	23.66	31.83
Standard deviation (SD)	14.89	7.68	7.38

Statistical analysis took place to determine whether there is a statistically significant difference in the percentage (%) change of TNBC cells in the "viable" and "dead" phases 24 hours after co-administration of different concentrations of docetaxel and dasatinib. The results prove that there is no significant difference between the two categories “viable” and “dead,” 24 hours after co-administration of different concentrations of docetaxel and dasatinib (p=0.11>0.05). In addition, an analysis to determine whether there is a statistically significant difference in the percentage (%) of TNBC cells in the "viable" and "apoptotic" phases 24 hours after co-administration of different concentrations of docetaxel and dasatinib also took place. The results suggest that there is a significant difference between the two categories (viable and apoptotic cells) 24 hours after co-administration of different concentrations of docetaxel and dasatinib (p=0.02<0.05).

Based on the above results, it appears that co-administration of docetaxel and dasatinib in TNBC cell lines induces/activates the process of programmed cell death (apoptosis) 24 hours after administration at a rate judged to be statistically significant (p<0.05) at all administered concentrations, with also a significant degree and profile of cytotoxicity, reflected by the increased number of dead cells. In fact, as the concentrations of these two administered drug agents (docetaxel and dasatinib) increased, the number of apoptotic and dead cells also increased.

Docetaxel and Dasatinib at 48 Hours

**Figure 8:**
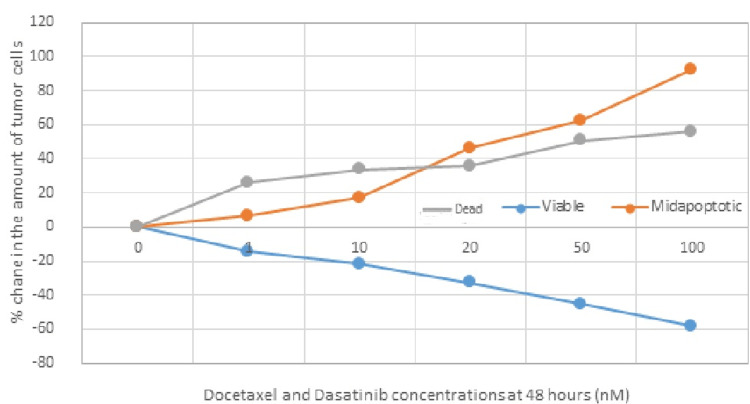
Graphical representation of the % change in the number of tumor cells 48 hours after administration of docetaxel and dasatinib at different concentrations of these pharmaceutical agents. nM: nanomoles; %: percentage

The following tables (Tables 11-12) and Figure 8 show the concentrations of administered docetaxel and dasatinib, as well as the number of viable, apoptotic, and dead cells in percentages (%), 48 hours after administration of different concentrations of these two drug agents in TNBC cell lines.

**Table 11:** The concentrations of the co-administered drug agents docetaxel and dasatinib and their effect on the percentage (%) of the number of cancer cells 48 hours after administration.

Doxetaxel and dasatinib concentration at 48 hours (nM)	Viable cells (%)	Apoptotic cells (%)	Dead cells (%)
C	55	18.8	26.2
1	47	20	33
10	43	22	35
20	37	27.5	35.5
50	30	30.5	39.5
100	23	36.2	40.8

**Table 12:** The mean and standard deviation of the % of TNBC cells in their different phases (viable, apoptotic, dead) 48 hours after co-administration of different concentrations of docetaxel and dasatinib.

	Viable cells (%)	Apoptotic cells (%)	Dead cells (%)
Mean	39.16	25.83	35
Standard deviation (SD)	11.63	6.78	5.20

Statistical analysis was made to determine whether there is a statistically significant difference in the percentage (%) change of TNBC cells in the "viable" and "dead" phases 48 hours after co-administration of different concentrations of docetaxel and dasatinib. Based on the results, there is no statistically significant difference between the two categories of cell phases (p=0.44>0.05). In addition, statistical analysis to determine whether there is a statistically significant difference in the % change in the percentage of TNBC cells in the "viable" and "apoptotic" phases 48 hours after co-administration of different concentrations of docetaxel and dasatinib showed that there is a statistically significant difference between the two categories ("viable" and "apoptotic"), 48 hours after the co-administration of different concentrations of docetaxel and dasatinib (p=0.041<0.05). Based on the above results, it appears that co-administration of docetaxel and dasatinib in TNBC cell lines induces/activates the process of programmed cell death (apoptosis) 48 hours of administration at a rate judged to be statistically significant at all administered concentrations, which comes along also with a significant degree of cytotoxicity, that is reflected by the increased number of dead cells. In fact, as the concentrations of these two administered drug agents (docetaxel and dasatinib) increased, the number of apoptotic and dead cells also increased.

The mean values of the body weight of the laboratory animals divided into groups are shown in the following tables (Tables 13-14).

**Table 13:** Mean values of the body weight of the laboratory animals of group A (control) and group B (DOC) before, during, and after the administration of docetaxel, until the animals were sacrificed.

Days	Group Β (mean of body weight)	Group A (mean of body weight)
6	23.06667	22.06667
9	23.13333	22.41667
11	23.03333	21.53333
13	23.9	23.06667
16	23.58333	22.86667
19	23.46667	22.43333
22	22.93333	22.5
25	23.31667	22.93333
28	23.56667	23.05
31	23.03333	23.95
33	23.03333	22.31667
36	21.86667	24.18333
40	20.98333	23.51667
47	17.62	22.33333
50	18	23.01667
53	17.275	22.9
56	16.1	23.16667
59	15.05	22.15
62	16.475	22.2
65	17.6	22.45
68	18.15	21.86667
69	Sacrifice	Sacrifice

**Table 14:** Mean values of the body weight of the laboratory animals of group C (DAS) and group D (DAS + DOC) before, during, and after the administration of docetaxel and dasatinib, until the animals were sacrificed. DOS: Docetaxel; DAS: Dasatinib

Days	Group C (mean of body weight)	Group D (mean of body weight)
5	21.88333	21.2
8	22.13333	21.6
11	22.78333	22.61667
14	22.25	21.5
17	21.48333	21.12
20	22.3	21.2
23	22.43333	22.9
42	23.61667	23.48
48	22	20.6
51	20	19.04
54	19.5	18.98
57	18.4	21.14
60	18.2	21.48
63	19.7	21.52
66	20.4	22.2
69	21.3	24.18
72	22.6	24.24
75	23.3	24.24
78	23.1	24.14
81	23.2	23.96
84	23.7	24.1
88	Sacrifice	Sacrifice

The effect of these agents/factors on body weight, adverse events, reduction in tumor size, and possible histological sub-staging was studied.

Docetaxel was administered by injection to the laboratory animals and the adverse effects that occurred included hair loss at the injection site, anorexia, and weight loss, while three mice of this group died. Dasatinib was administered via the food to the laboratory animals and had no clinically observed adverse effects. The effect on tumor growth and body weight of the laboratory animals of the co-administration of the two agents was also studied, which has not been studied at the clinical level so far.

**Figure 9:**
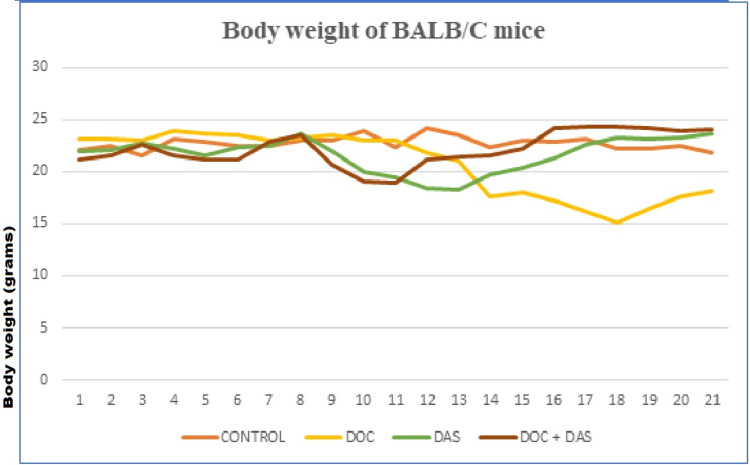
Summary plot of the variation in the mean body weights of laboratory animals (BALB/c hairless athymic mice) in the four groups of the in vivo experimental procedure. DOC: Docetaxel; DAS: Dasatinib

Figure 9 reveals the variation in body weight of the laboratory animals from the day of tumor cell inoculation (day one) to the day of sacrifice.

**Figure 10:**
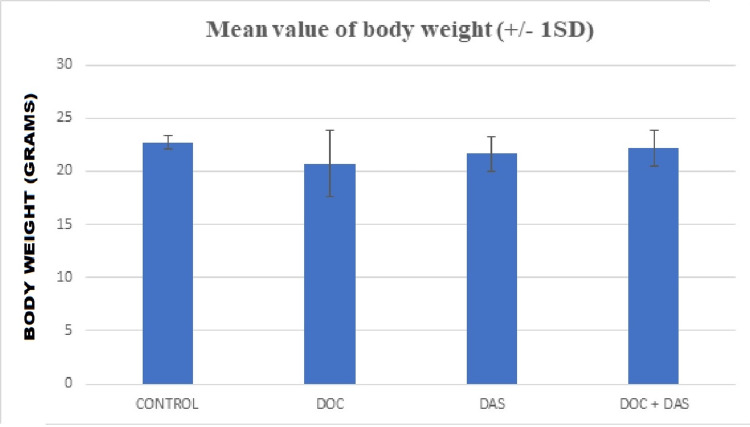
Bar chart representation of the mean body weights of laboratory animals with the standard deviation (+/- 1SD) plotted as an error bar at the top of each column. DOC: Docetaxel; DAS: Dasatinib

**Figure 11:**
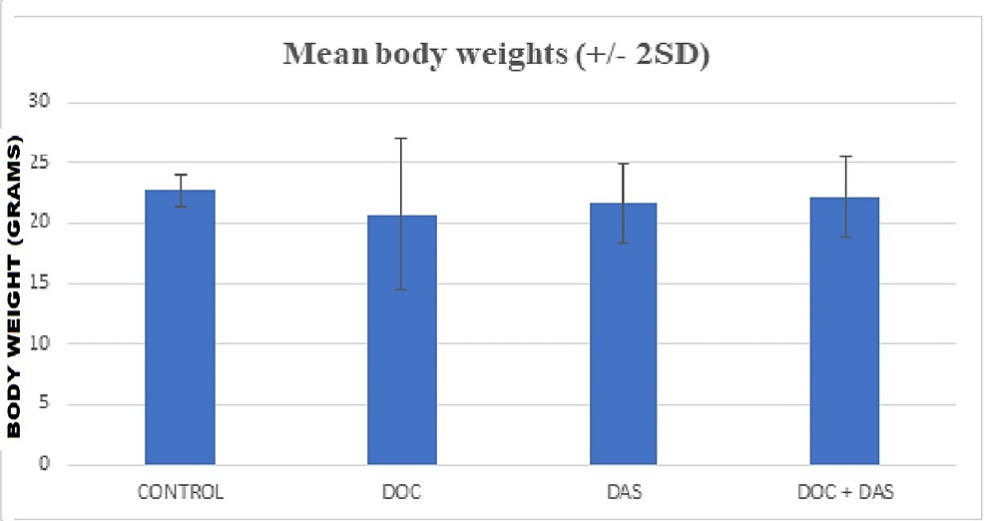
Bar chart representation of the mean body weights of laboratory animals, with the two standard deviations (+/- 2SD) plotted as error bars at the top of each column. DOC: Docetaxel; DAS: Dasatinib

The following figures (Figures 10-11) depict the mean values of the body weight of the four groups that participated in the in vivo experimental procedure, with the standard deviations shown as error bars at the top of the columns.

A further statistical analysis was performed to determine whether or not there was a statistically significant difference in body weight change between the control group and each of the other three groups in the experiment separately, as well as between group C (DAS) and group D (DAS + DOC).

Comparison group A (control) - group B (Docetaxel): Statistical analysis to determine whether there is a statistically significant difference in body weight change between the control group and laboratory animals receiving docetaxel (DOC) was made. Results of the statistical analysis reveal that there is a statistically significant decrease in body weight between the laboratory animals of the control group (group A) and those receiving docetaxel (group B) (p=0.008<0.05).

Comparison group A (control) - group C (Dasatinib): Statistical analysis to determine whether there is a statistically significant difference in body weight change between the control group and laboratory animals receiving dasatinib (DAS) was made. The results reveal that there is a statistically significant decrease in body weight between the laboratory animals of the control group (group A) and those receiving dasatinib (group C) (p=0.01<0.05).

Comparison group A (control) - group D (Docetaxel + Dasatinib): Statistical analysis to determine whether there is a statistically significant difference in body weight change between the control group and laboratory animals treated with a combination of docetaxel and dasatinib was realized. Results conclude that there is no statistically significant difference in body weight change between the laboratory animals in the control group (group A) and those receiving a combination of docetaxel and dasatinib (group D) (p=0.16>0.05).

Comparison group C (Dasatinib) - group D (Docetaxel + Dasatinib): Statistical analysis to determine whether there is a statistically significant difference in the change in body weight between the group receiving dasatinib and the laboratory animals receiving a combination of docetaxel and dasatinib took place.

The results conclude that there is no statistically significant difference in the change of body weight between the laboratory animals of the group receiving dasatinib (group C) and those receiving a combination of docetaxel and dasatinib (group D) (p=0.3>0.05).

It thus appears that the decrease in body weight among the laboratory animals was statistically significant in the groups receiving injectable docetaxel and oral via dietary dasatinib when these groups were compared with the control group. The DOC group featured the highest weight body loss, which may be related to its cytotoxicity. It should be noted that despite the fact that palpable masses were found in the mammary glands, specifically in the areas of inoculation of TNBC cell lines, it was not possible to find a histological-histopathological tumor with TNBC cancer cells.

## Discussion

TNBC has the worst prognosis of all molecular BC subtypes, with much shorter disease-free survival and outcome survival intervals [25]. It appears that TNBC has both an intrinsic and acquired resistance to chemotherapy, which leads to disease recurrence and the development of metastases, despite an initial response to cytotoxic chemotherapy with taxanes and platins. The discovery of an abundance of primitive-differentiated BC cells or BC stem cells (BCSCs) in TNBC cell lines seems to be a major contributor to the aforementioned resistance and failure of chemotherapy [26]. Specifically, dasatinib reduces BCSC numbers by inhibiting taxane (paclitaxel)-induced activation of Src kinase. Moreover, epithelial-to-mesenchymal transition (EMT) appears to enrich neoplastic cells with properties of primordial, undifferentiated cells. Dasatinib was observed to induce phenotypic epithelial differentiation of BCSCs [26]. The differentiated cells are sensitive to chemotherapy, so the administration of dasatinib and paclitaxel exhibits a synergistic effect in reducing cell viability of chemoresistant cancer cells in vitro and reducing tumor volume in vivo, thereby overcoming the resistance to cytotoxic drug chemotherapy that characterizes TNBC [26].

In a study published in 2019, Gaule et al. observed that the combined administration of dasatinib and C-met inhibitor (CpdA) induces inhibition of growth/cell proliferation of MDA-MB-231 cell lines in vitro, preventing the development of tolerance/resistance to dasatinib activity [27]. They report that these findings could be extrapolated to in vivo experimental protocols, giving hope for more effective treatment of patients with TNBC [27]. In two other recent experimental studies implicating dasatinib with TNBC, it is emphasized that the early onset of treatment resistance and the absence of typical biomarkers (hence the absence of targeted therapies) are the two most important factors in determining the poor prognosis of TNBC [28]. However, they also incorporate another factor that seems to play a role in removing initial efficacy. Dasatinib, as previously reported, is a kinase inhibitor that has already received approval for administration in patients with TNBC. Its oral administration, though, leads to its binding to plasma proteins, resulting in extensive metabolism via acidification and coupling events that ultimately result in pharmacokinetic degradation and prolongation of its action [29]. It appears that the rapid absorption and 80% bioavailability of dasatinib disappears after being metabolized by CYP3A4, with its half-life estimated at 3-4 hours [29]. Src protein kinase, for its part, has been associated with carcinogenic events at the intracellular level, such as growth, survival, intracellular adhesion, and infiltration, as well as with VEGF expression. This kinase also indicates the metastatic potential of TNBC.

The Src kinase inhibitor dasatinib, which, at the preclinical level, inhibits the cellular proliferation of cancer cells, shows synergistic activity with chemotherapeutic drugs; in clinical practice, the results of its action are not as impressive. For this reason, encapsulation of dasatinib in nanoparticles was proposed and generated at the experimental level, resulting in a statistically significant improvement in its bioavailability and delay of pharmacokinetic degradation [28,29]. Finally, a very recent study by Moussa et al. in 2021 attempts to correlate two distinct signaling pathways, those of COX-2 and Src, which is a new and extremely interesting topic in the treatment of TNBC. The investigators administered dasatinib and celecoxib (a COX-2 inhibitor), as monotherapy and as a combination, to TNBC cell lines [30]. It appears that dasatinib caused inhibition of Src kinase expression at the protein level (indirectly upregulation of the c-Src gene expression level), suppression of AKT phosphorylation, an increase in the expression of caspase-3 (which induces apoptosis), a decrease in the levels of cyclin D1 protein (hence cell cycle arrest in G1 phase), and a decrease in VEGF levels [31]. Celecoxib exhibited similar or equivalent actions, supporting the hypothesis of commonalities in the signaling pathways of these two agents [30,31].

Regarding the in vitro experimental procedure, triple-negative MDA-MB 231 BC cell lines were treated with the drug agents docetaxel and dasatinib, alone and in combination. Their effect on both growth inhibition and apoptosis induction at both 24 and 48 hours for their various concentrations was studied. From the processing of the results, it appears that the administration of docetaxel to TNBC cell lines induces/activates the process of programmed cell death (apoptosis) 24 hours after administration at a rate that is judged to be statistically significant, at all administered concentrations, with also a significant degree and profile of cytotoxicity, reflected by the increased number of dead cells. Indeed, as the concentrations of administered docetaxel increased, the number of apoptotic and dead cells also increased. The administration of docetaxel to TNBC cell lines induces/activates the process of programmed cell death (apoptosis) 48 hours after administration at a rate judged to be statistically significant, at all administered concentrations, while the degree of cytotoxicity, reflected by the number of dead cells, does not appear to be as pronounced as at 24 hours. Additionally, the administration of docetaxel at increasing concentrations correspondingly increases the degree of apoptosis at 48 hours, with no significant effect on the number of dead cells. In fact, as the concentrations of docetaxel administered increased, the number of apoptotic cells also increased.

Based also on the above results, it appears that the administration of dasatinib in TNBC cell lines induces/activates the process of programmed cell death (apoptosis) 24 hours after administration at a rate that is judged to be statistically significant, at all administered concentrations, with also a significant degree and profile of cytotoxicity, reflected by the increased number of dead cells. Indeed, as the concentrations of administered dasatinib increased, the number of apoptotic and dead cells also increased. Meanwhile, administration of dasatinib at various increasing concentrations induces programmed cell death (apoptosis) 48 hours after administration to a statistically significant degree with a low degree of cytotoxicity. Indeed, apoptosis increases with increasing concentrations of the administered drug in the cellular tumor lines, without a significant effect on the number of dead/killed cells. Furthermore, co-administration of docetaxel and dasatinib in TNBC cell lines induces/activates the process of programmed cell death (apoptosis) 24 hours after administration at a rate judged to be statistically significant at all administered concentrations, with also a significant degree and profile of cytotoxicity, reflected by the increased number of dead cells. In fact, as the concentrations of these two administered drug agents (docetaxel and dasatinib) increased, the number of apoptotic and dead cells also increased. Finally, co-administration of docetaxel and dasatinib in TNBC cell lines induces/activates the process of programmed cell death (apoptosis) 48 hours after administration at a rate judged to be statistically significant at all administered concentrations, with also a significant degree and profile of cytotoxicity, reflected by the increased number of dead cells. In fact, as the concentrations of these two administered drug agents (docetaxel and dasatinib) increased, the number of apoptotic and dead cells also increased. There is only one study to date investigating the effect of the agent's docetaxel and dasatinib on TNBC cell lines. It concludes that co-administration of 100 nM dasatinib with various concentrations of docetaxel has a synergistic effect on MDA-MB 231 TNBC cell lines [32]. These results agree with the results of the present study.

When it comes to the in vivo experimental procedure, clinically perceivable tumor-BC was induced in hairless, athymic BALB/c laboratory animals. These were then divided into four groups and administered the pharmaceutical agents (docetaxel and dasatinib). The effect of these agents on body weight, the induction of adverse effects, during the in vivo experimental procedure, and clinically perceived tumor-BC was induced in hairless, athymic BALB/c laboratory animals. These were then divided into four groups and administered the pharmaceutical agents (docetaxel and dasatinib). The effect of these agents on body weight, adverse effects, reduction in tumor size, and possible histological hypostasis were studied [33]. Docetaxel was administered by injection to the laboratory animals and caused adverse effects, which were hair loss at the injection site, anorexia, and weight loss, while three mice died in this group. There was a statistically significant reduction in tumor size in the laboratory animals that survived the cytotoxicity of the chemotherapeutic. Dasatinib was administered to the laboratory animals through diet and had no clinically observed adverse effects. It had a statistically significant effect on body weight change (reduction) compared to the control group, with no appreciable profile of clinically perceived adverse effects. The effect on tumor growth and body weight of laboratory animals of the co-administration of the two agents was also studied. These effects have not been studied at the clinical level so far. The synergistic effect of the two agents in the in vivo experimental procedure demonstrated a non-statistically significant reduction in the body weight of the laboratory animals and an acceptable profile of adverse effects. The findings from the in vivo experiment could be a reason to initiate similar studies to further prove their synergistic action, as they seem to have less effect on the laboratory animals. Further studies will investigate the possible histological downstaging of TNBC in xenograft models in vivo [34-38].

Some observations regarding the limitations of the study include the following: There was a difference in the pharmaceutical form of dasatinib (oral administration - tablet) and docetaxel (formulation for intravenous administration). In the in vivo experimental procedure, the sample (4x6=24 BALB/c athymic laboratory hairless animals) could be considered as limited, while the administration of the pharmaceutical agents to be tested via food (dasatinib) resulted in an alteration (attenuation) of the pharmacokinetics of dasatinib, which could potentially affect the comparison with docetaxel. Additionally, the lack of histological TNBC tumor development is a major drawback of the present study, although there was mass gland hyperplasia in the laboratory animals.

## Conclusions

Although TNBC is a challenging oncologic condition to manage even in an experimental setting, the combination of targeted medications (dasatinib) and conventional cytotoxic therapies (docetaxel) has shown favorable outcomes. According to the findings of this study, combining the aforementioned agents can induce apoptosis in TNBC cell lines in 24 and 48 hours while retaining an acceptable cytotoxicity profile. Dasatinib plus docetaxel, when administered separately in xenograft TNBC models, results in a moderate body weight and tumor size reduction, with countable adverse drug reactions. More basic and transitional research, however, is required to corroborate the current study's findings and further clarify the underlying mechanisms, in order to further support the use of targeted therapy in daily clinical practice against TNBC.
